# Changes in the stoichiometry of *Castanopsis fargesii* along an elevation gradient in a Chinese subtropical forest

**DOI:** 10.7717/peerj.11553

**Published:** 2021-06-01

**Authors:** Danping Liu, Dexiang Zheng, Yaoyao Xu, Yifei Chen, Hesong Wang, Ku Wang, Xiaoli Liao, Changxiong Chen, Jiangjiang Xia, Shaofei Jin

**Affiliations:** 1 College of Forestry, Fujian Agriculture and Forestry University, Fuzhou, China; 2 School of Ecology and Nature Conservation, Beijing Forestry University, Beijing, China; 3 Department of Geography, Minjiang University, Fuzhou, China; 4 Key Laboratory of Regional Climate-Environment for Temperate East Asia, Institute of Atmospheric Physics, Chinese Academy of Sciences, Beijing, China; 5 Institute of Eco-Chongming, East China Normal University, Shanghai, China

**Keywords:** Global warming, Subtropical evergreen broad-leaved forest, Elevation, *Castanopsis fargesii*, Leaf stoichiometry

## Abstract

Elevation is important for determining the nutrient biogeochemical cycle in forest ecosystems. Changes in the ecological stoichiometry of nutrients along an elevation gradient can be used to predict how an element cycle responds in the midst of global climate change. We investigated changes in concentrations of and relationships between nitrogen (N), phosphorus (P), potassium (K), calcium (Ca), and magnesium (Mg) in the leaves and roots of the dominant tree species, *Castanopsis fargesii,* along an elevation gradient (from 500 to 1,000 m above mean sea level) in a subtropical natural forest in China. We analyzed correlations between *C. fargesii*’s above-ground biomass and stoichiometry with environmental factors. We also analyzed the soil and plant stoichiometry of this* C. fargesii* population. Our results showed that leaf N decreased while leaf K and Ca increased at higher elevations. Meanwhile, leaf P showed no relationship with elevation. The leaf N:P indicated that *C. fargesii* was limited by N. Elevation gradients contributed 46.40% of the total variance of ecological stoichiometry when assessing environmental factors. Our research may provide a theoretical basis for the biogeochemical cycle along with better forest management and fertilization for this *C. fargesii* population.

## Introduction

The global forest ecosystem is affected by increasing global temperatures caused by excess greenhouse gases from anthropogenic activities (Wang et al., 2016). Global warming has affected nutrient element cycling in forest ecosystems (Penuelas & Matamala, 1990; Penuelas & Matamala, 1993; Penuelas et al., 2020; Sardans et al., 2015) and its impact on the biogeochemical cycle is difficult to ignore. Global warming may alter the distribution of nutrient elements by impacting plant metabolism, thus affecting nutrient transfer in plant organs (Gavito et al., 2005; Jónsdóttir, Khitun & Stenström, 2005; Yan, Zhu & Yang, 2017). Two the large-scale effects of global warming on element cycling have been studied to date: latitudinal effects over large areas (He et al., 2006; Han et al., 2011; Ordoñez et al., 2009; Zhang et al., 2012; Fang et al., 2019) and altitude effects. These are crucial factors for determining how variations in temperature and relative climatic changes drive ecological processes (Körner, 2007; Normand et al., 2009).

Recently, studies on plant stoichiometry changes along elevation gradients have not shown uniform patterns of change. Changes in plant N has shown an increase (Richardson, Berlyn & Gregoire, 2001; Shi, Körner & Hoch, 2006), decrease (Li & Sun, 2016; Soethe, Lehmann & Engels, 2008; Van De Weg et al., 2009), or no linear correlation (Macek et al., 2012) with increasing elevation. Most studies have shown that plant P decreased with increasing elevation (Soethe, Lehmann & Engels, 2008; Tanner, Vitousek & Cuevas, 1998; Vitousek et al., 1992; Wang et al., 2018). Elevational gradients may lead to significant variations in regional microclimate and soil properties (Zeng et al., 2018; Chang et al., 2016) , which further affects the nutrient cycling of a plant-soil system in forest ecosystems. This suggests that there is closely-coupled element cycling between C, N, and P in the plant-soil system (Elser et al., 2000). Deng et al. (2008) demonstrated that plant P had a significant positive correlation with soil P in the subtropical forest karst area. Plant stoichiometry was also affected by environmental factors. Studying plant stoichiometry and its correlation with environmental factors may provide a theoretical understanding of plants’ nutritional requirements and their mutual feedback with the environment (Li et al., 2019). Reich & Oleksyn (2004) showed that leaf N and P increases from the tropics to mid-latitudes due to temperature-related plant physiological stoichiometry and soil substrate age; these elements decreased at higher latitudes because cold temperatures affect biogeochemistry. Ma et al. (2015) found that root N and P were negatively correlated with annual average temperature and annual precipitation. Many studies of plant stoichiometry have focused on C, N, and P but few have considered the stoichiometry of other crucial elements, including K, Ca, and Mg.

*Fagaceae* is a vital subtropical plant in China that is important for maintaining and promoting the element cycling of subtropical forest ecosystems. Zheng et al. (2017) showed that there were significant differences between leaf and root C, N, and P in seedlings and young trees of *Castanopsis fissa* and other plant organs. Chang et al. (2013) showed that P was the most important element for limiting plant productivity in the subtropical forest ecosystem. *Castanopsis fargesii* is one of the main species of the subtropical forests of China; however, there is little information on its stoichiometry, which affects the regional forest ecosystem and element cycle characteristics. Previous studies have focused on the ecological characteristics of *C. fargesii*, including its community structure, photosynthetic characteristics, community biomass, and soil organic carbon (Song et al., 2003; Zhao et al., 2005; Qian et al., 2004; Gong et al., 2015; Dai et al., 2018). However, changes *C. fargesii* ’s stoichiometry along elevation gradients have not been well-studied (Liu et al., 2019; Dai et al., 2018).

Temperatures are known to change with increasing elevation, even over short distances (Tan & Wang, 2016; Paudel et al., 2019). The elevational changes in temperature and soil nutrients may have an effect on plant stoichiometry (Normand et al., 2009; Sundqvist, Sanders & Wardle, 2013; Yu et al., 2013). Thus, we conducted experiments with five elevation gradients as a proxy for the effects of global warming in a subtropical natural forest on Guoyan Mountain in southern China. We sought to investigate the plant (specifically in its leaves and roots) and soil stoichiometry (N, P, K, Ca, and Mg) in *C. fargesii* populations along elevation gradients and determine the relative importance of elevation to the total variation of ecological stoichiometry.

## Material and Methods

### Study area description

**Figure 1:**
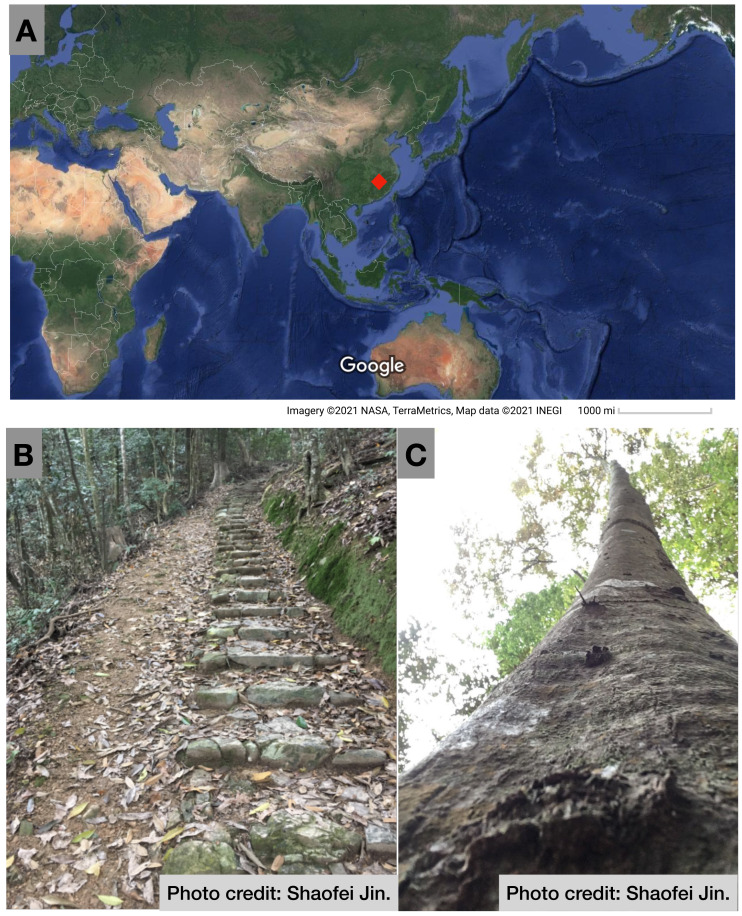
(A) Location of this study, (B) Ancient road in the Guoyan Mountain Natural Reserve, and (C) Photo of *Castanopsis fargesii.* Photo credit: Shaofei Jin. Map data ©2021 Google.

The study was conducted in the Guoyan Mountain Natural Reserve on Wuyi Mountain (17°29′ ∼118°14′E, 26°38′ ∼27°12′N) in the northwestern Fujian Province. Its peak elevation was 1,383.7 m (Fig. 1). The study area had a moderate-subtropical monsoon climate with an average annual temperature of 19 °C and an average annual rainfall of 2,051 mm. *C. fargesii* was one of the dominant species along the elevation gradients in the study area, which ranged from 500 to 1,000 m.

### Sampling

Elevation gradients were established from 500 to 1,000 m and covered *C. fargesii*’s natural habitat. Forests were intentionally planted below this range according to our field observations. Five 30 m × 30 m plots were established at 100 m intervals (i.e., 500–600 m, 600–700 m, 700–800 m, 800–900 m, and 900–1,000 m, respectively, which were denoted as 500 m, 600 m, 700 m, 800 m, and 900 m). Three plots were established at 500 m where fewer *C. fargesii* were found and are close to bamboo plantations. Five *C. fargesii* specimens were selected randomly in each plot and leaf samples were collected from each tree that exhibited good growth conditions. Root samples were collected from the same five trees. Soil samples were collected from around each tree at three depths (0–20 cm, 20–40 cm, and 40–60 cm) using a soil sampling auger with a diameter of five cm. All samples from each plot were mixed thoroughly and stored at 4 °C before being transported to a laboratory. The specimens’ biodiversity index, height, and diameter at breast height (DBH) were also recorded (Table 1).

### Laboratory analyses

Roots were deposited into 0.15 mm net bags and washed under running water. Fine roots (d < 2 mm) were separated. The leaves and roots were cleaned with distilled water and dried at 50 °C to a constant weight. All plant parts were ground prior to analysis. We removed stones and visible litter from the soil samples and then air-dried and sieved the soil through a two mm nylon mesh. We used 0.10 g of leaf and root material and 0.20 g of soil material to determine the N content using a Vario Max CN analyzer (Elementar, Germany). The leaf and soil P, K, Ca, and Mg concentrations were determined using Inductively Coupled Plasma- Mass Spectrometry (ICP-MS) (PE Optima 8000) following H_2_SO_4_/HClO_4_ and HF/HClO_4_ digestion.

**Table 1:** Importance value index, DBH, and tree height of *C. fargesii* at different elevations (*n*  = 15).

Elevation (m)	Importance value index	DBH (cm)	Tree height (m)
400	0	NA	NA
500	38.70	15.50 ± 1.15	12.33 ± 0.76
600	43.46	17.06 ± 2.19	14.40 ± 0.80
700	26.09	14.22 ± 2.36	12.30 ± 3.99
800	40.53	13.00 ± 1.99	9.80 ± 2.22
900	71.12	10.58 ± 1.04	9.80 ± 0.94
1,000	0	NA	NA

**Notes.**

DBH diameter at breast height NA not available.

### Estimating above-ground biomass of *C. fargesii*

The above-ground biomass (AGB) of *C. fargesii* in each plot was estimated according to the *2006 IPCC Guidelines for National Greenhouse Gas Inventory* (Eggleston et al., 2006). AGB was obtained as follows: Eqs. (1) and (2)

(1) \begin{eqnarray*}& \text{AGB}=V\times n\times S\div s\times BCEFs\end{eqnarray*}

(2) \begin{eqnarray*}& V={g}_{\mathrm{1.3}}\times (H+3)\times {f}_{3}\end{eqnarray*}

where *V* is the volume of an individual *C. fargesii*; *g*_1.3_ is the basal area of breast-height; *H* is the tree height; *f*_3_ is the experimental form factor; the value of the broad-leaved trees is 0.40 (Meng, 2006); *S* is the 1 hm^2^ of the stand area; *s* is the stand area of each plot; *BCEFs* is the biomass conversion and expansion factor of growing-stock. Its value is 0.66 in subtropical regions (Eggleston et al., 2006).

### Statistical analysis

Leaf, root, and soil stoichiometry variations at different elevations were compared using one-way ANOVA. Multiple comparisons were performed using Tukey-HSD post hoc tests. The correlations between the environmental factors, plant tissues, and soil stoichiometry of the *C. fargesii* community were determined using redundancy analysis (RDA). All data were checked to assess whether they met the assumptions of homogeneity and normality. All analyses and figures were determined at a significance level of *p* < 0.05 using R software. All raw data are shown in Supplemental File 1.

## Results

### Variations in the plant stoichiometry of *C. fargesii* along the elevation gradients

**Figure 2:**
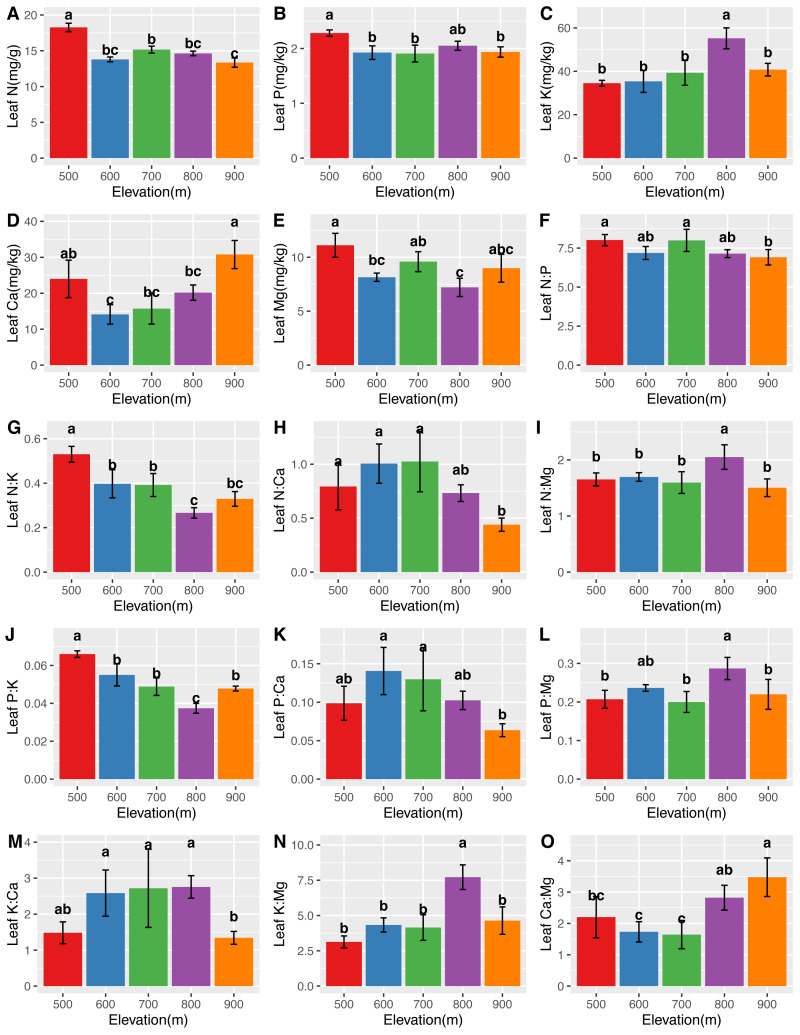
Variations in the leaf N, P, K, Ca, and Mg concentrations (A–E) and the leaf N:P, N:K, N:Ca, N:Mg, P:K, P:Ca, P:Mg, K:Ca, K:Mg, and Ca:Mg ratios (F–O) at different elevations. The error bars represent standard deviation. Different letters indicate that there are significant differences among different elevations (*p* < 0.05).

**Figure 3:**
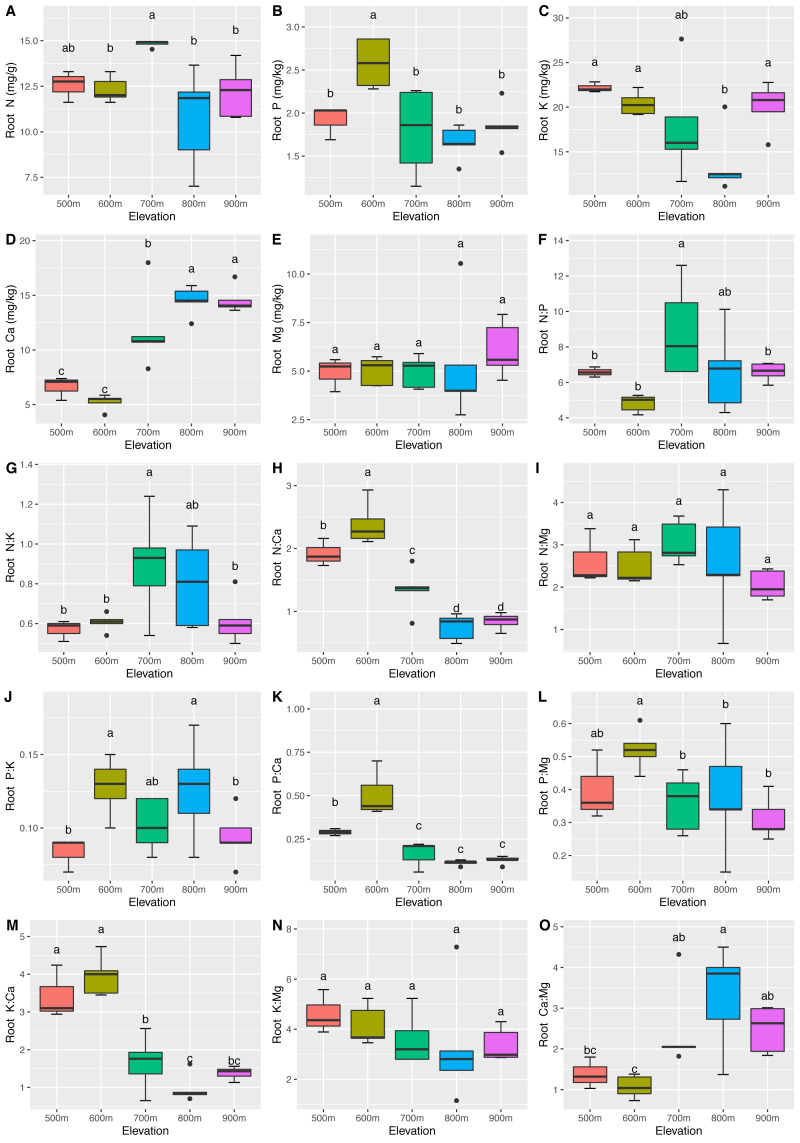
Variations in the root N, P, K, Ca, and Mg concentrations (A–E) and the root N:P, N:K, N:Ca, N:Mg, P:K, P:Ca, P:Mg, K:Ca, K:Mg, and Ca:Mg ratios (F–O) at different elevations. The error bars represent standard deviation. Different letters indicate that there are significant differences among different elevations (*p* < 0.05).

The mean N, P, K, Ca, and Mg concentrations in the leaves were 14.76 ± 1.60 mg/g, 2.00 ± 0.16 mg/kg, 41.59 ± 8.71 mg/kg, 20.68 ± 7.14 mg/kg, and 8.83 ± 1.51 mg/kg, respectively. The mean N, P, K, Ca, and Mg concentrations in the roots were 12.53 ± 1.91 mg/g, 1.96 ± 0.43 mg/kg, 18.56 ± 4.29 mg/kg, 10.89 ± 4.29 mg/kg, and 5.30 ± 1.56 mg/kg, respectively. Significant differences (*P* <0.05) in the leaf (Fig. 2) and root (Fig. 3) ecological stoichiometry were found among different elevation gradients. The linear regressions between the ecological stoichiometry and the elevation gradients are shown in Fig. S1 and Fig. S2, respectively. The greatest leaf N concentration was found at the lowest elevation. The mean leaf N:P along the elevation was 7.50 ± 0.63. A significantly positive correlation was found between leaf K and elevation. The leaf and root Ca increased significantly at higher elevations.

### Correlations between AGB and plant stoichiometry

**Figure 4:**
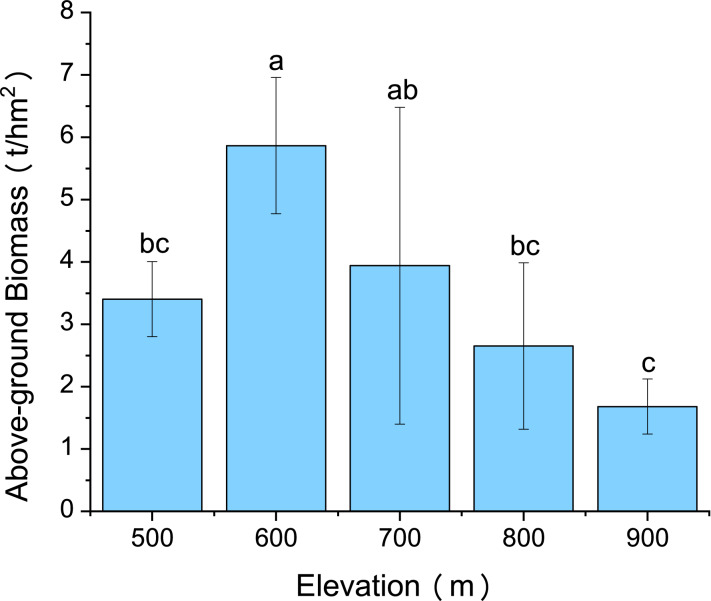
Changes in the above-ground biomass of *C. fargesii* along the elevation gradients. The error bars represent the standard deviation. Different letters indicate that there are significant differences among different elevations (*p* < 0.05).

**Figure 5:**
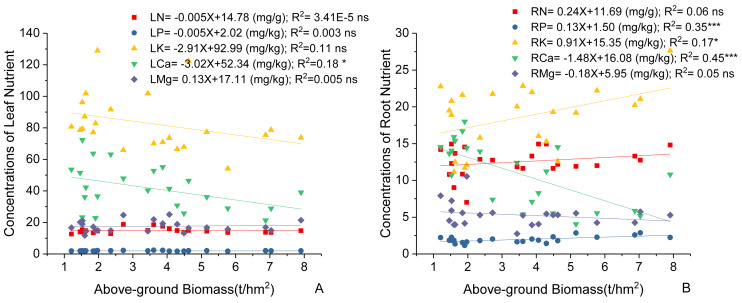
Linear regression between (A) leaf nutrients, (B) root nutrients (N, P, K, Ca, Mg) and above-ground biomass of the *C. fargesii*. LN, leaf N concentrations; LP, leaf P concentrations; LK, leaf K concentrations; LCa, leaf Ca concentrations; LMg, leaf Mg concentrations; RN, root N concentrations; RP, root P concentrations; RK, root K concentrations; RCa, root Ca concentrations; RMg, root Mg concentrations. ns, not significant, *: *p* < 0.05, **: *p* < 0.01, ***: *p* < 0.001.

Variations in *C. fargesii*’s AGB at different elevations are shown in Fig. 4. The greatest AGB was found at 600 m. The AGB showed a significantly negative correlation with leaf Ca and no correlation with other leaf stoichiometry. The AGB also showed a significantly positive correlation with root K and root P. There was a significantly negative correlation between the AGB and root Ca (Fig. 5).

### Relationships between soil stoichiometry and plant stoichiometry in the *C. fargesii* population

**Figure 6:**
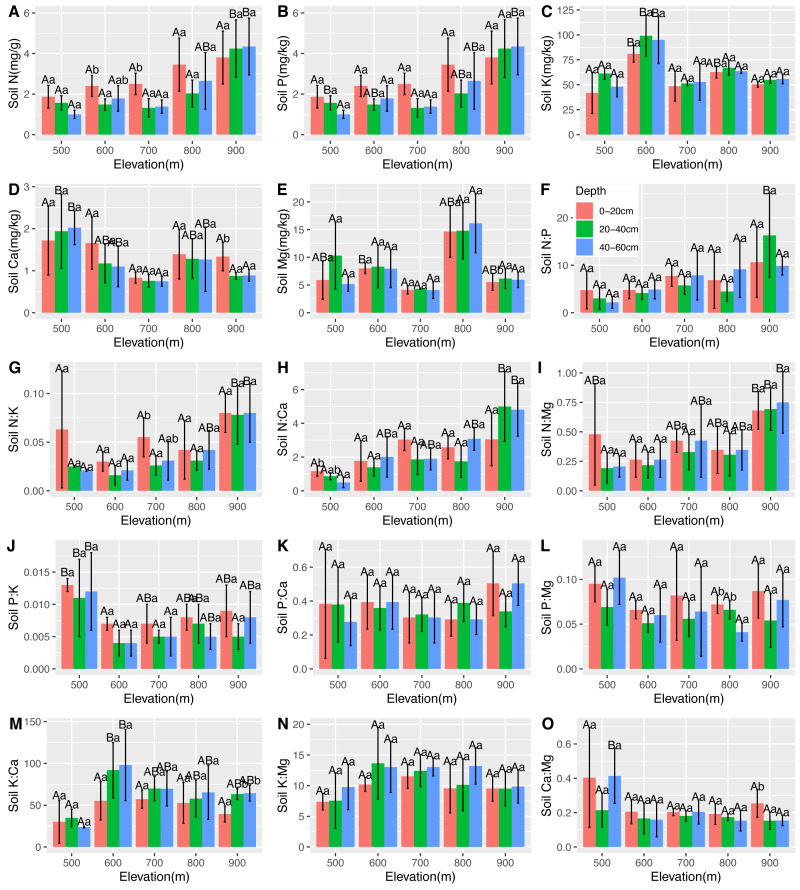
Variations in the soil N, P, K, Ca, and Mg contents (A–E) and the soil N:P, N:K, N:Ca, N:Mg, P:K, P:Ca, P:Mg, K:Ca, K:Mg, and Ca:Mg ratios (F–O) at different elevations and soil depths. The error bars represent standard deviation. Different uppercase letters within the panels indicate that there are significant differences between different elevations (*p* < 0.05), and different lowercase letters within the panels indicate that there are significant differences between different soil depths (*p* < 0.05).

There were significantly positive correlations between soil N, leaf Ca, and root Ca. There were significantly negative correlations between soil N, leaf N, and leaf Mg. However, there was no correlation between soil P and leaf and root nutrients. Soil K was found to be positively correlated with root P and negatively correlated with leaf Ca, leaf Mg, and root Ca. Soil Ca was found positively correlated with leaf N and leaf P. There was a significantly positive correlation between soil Mg and leaf K, and a negative correlation between soil and leaf Mg, root N, and root K (Table 2). The changes in the soil stoichiometry of the *C. fargesii* community are shown in Fig. 6.

**Table 2:** Correlation analysis between soil stoichiometry and plant stoichiometry of *C. fargesii*.

Soil stoichiometry	Leaf stoichiometry					Root stoichiometry				
	Leaf N	Leaf P	Leaf K	Leaf Ca	Leaf Mg	Root N	Root P	Root K	Root Ca	Root Mg
Soil N	−0.46^***^	−0.16	0.17	0.44^***^	−0.21^*^	−0.14	−0.056	0.028	0.4^***^	0.012
Soil P	0.059	0.11	−0.048	0.2	0.026	−0.085	0.14	0.081	0.064	−0.0035
Soil K	−0.29^*^	−0.051	−0.011	−0.34^**^	−0.35^**^	−0.22^*^	0.66^***^	0.059	−0.48^***^	−0.00094
Soil Ca	0.36^**^	0.37^**^	−0.036	0.065	0.03	−0.25^*^	0.15	−0.024	−0.24^*^	−0.12
Soil Mg	−0.068	0.2	0.62^***^	−0.037	−0.52^***^	−0.35^**^	−0.0079	−0.49^***^	0.21^*^	−0.091

**Notes.**

* *p* < 0.05.

** *p* < 0.01.

*** *p* < 0.001

No asterisk denotes no significant correlation coefficient was found.

### Relationships between environmental factors and leaf and root nutrients in the *C. fargesii* population

**Figure 7:**
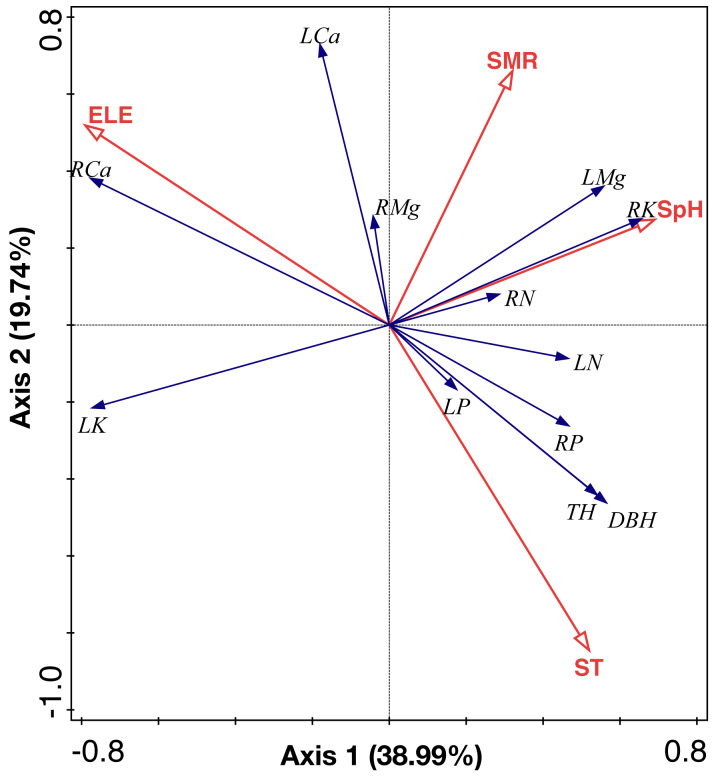
RDA analysis of environmental factors, leaf and root nutrient of the *C. fargesii* community in the Guoyan Mountain Natural Reserve. ST, soil temperature; ELE, elevation; SM, soil moisture; SpH, soil pH; LN, leaf N concentrations; LP, leaf P concentrations; LK, leaf K concentrations; LCa, leaf Ca concentrations; LMg, leaf Mg concentrations; RN, root N concentrations; RP, root P concentrations; RK, root K concentrations; RCa, root Ca concentrations; RMg, root Mg concentrations.

RDA analysis identified two main axes of environmental variation that together accounted for 58.73% of the total variance of ecological stoichiometry (Fig. 7, Tables 3 and 4). The first RDA axis contributed 38.99% of total variance which was explained by soil pH and elevation. The second RDA axis contributed 19.74% of total variance and was mainly explained by soil moisture and soil temperature. We show the ranking of environmental variables in the order of contribution in Table 3. Elevation, soil moisture, soil temperature, and soil pH contributed 46.4%, 33.8%, 16%, and 3.9%, respectively.

## Discussion

### Plant stoichiometry responses to elevation

We determined that the greatest leaf N concentration of *C. fargesii* was found at the lowest elevation (Fig. 2). This result was consistent with previous studies (Li & Sun, 2016; Soethe, Lehmann & Engels, 2008; Van De Weg et al., 2009). Lower temperatures were found at higher elevations, which decreased the decomposition and mineralization of organic matter (Hobbie et al., 2000; Hobbie, Nadelhoffer & Högberg, 2002; He et al., 2016) and thus decreased the availability of leaf N (Reich & Oleksyn, 2004). Leaf N is typically determined by plant growth characteristics, whereas leaf P is determined by plant growth and environment. Therefore, leaf N is more stable and consistent than leaf P (Chen et al., 2013). According to Güsewell (2004), leaf N:P <10 represents the N limitation, and N:P >20 represents the P limitation. *C. fargesii* was limited by N but not P, since N was lower than that in lower-elevation plants in China (Han et al., 2011; Zhang et al., 2012). There were significant differences in root N and P across elevations. Root N and P were greater than those of lower-elevation plants in China (Ma et al., 2015). There was a significantly positive correlation between leaf K and elevation, which was supported by the findings of Du et al. (2017). This indicates that the ecology at higher elevations did not restrict K uptake by plants. Leaf Ca increased with elevation except at 500 m. Root Ca increased at higher elevations. The lower temperatures at higher elevations promoted Ca uptake by plants, which aided in their defense against the cold (Plieth et al., 1999). *C. fargesii* required more Ca at higher elevations and we predict that its demands at the same elevation may decrease in the future with global warming and altered forest lines.

### Correlations between AGB and *C. fargesii* plant stoichiometry

*C. fargesii*’s highest AGB (Fig. 4) and importance value (Table 1) were found at 600 m, indicating that this environment is more suitable for growth than those at other elevations. However, the AGB decreased as elevation increased and species’ tendency to migrate to a higher elevation was not noticeable despite being affected by warming temperatures and the tree line moving up. Leaf and root Ca were significantly negatively correlated with *C. fargesii* AGB indicating that *C. fargesii* with a larger above-ground biomass had less demand for Ca. *C. fargesii* at a higher elevation may require more Ca than those at lower elevations in our research area. Root P and K were significantly positively correlated with AGB indicating that *C. fargesii* with a larger AGB had more demand for P and K. In general, P-limiting was found in the acidic soil of tropical and subtropical regions. However, we found that P-limiting did not exist in our research area and P was absorbed by plant roots as an essential nutrient in P-rich soils. Estimating the AGB of *C. fargesii* at different elevations and analyzing the relationship between AGB and the concentration of plant stoichiometry may provide a basis for *C. fargesii* management and fertilization in the future.

**Table 3:** Summary of environmental factor at different elevation gradients.

Elevation (m)	Soil temperature (° C)	Soil moisture (%)	Soil pH
500	17.13 ± 7.13	14.12 ± 4.12	5.34 ± .342
600	17.93 ± 7.93	15.98 ± 5.98	4.73 ± .738
700	16.40 ± 0.36	14.02 ± 4.02	4.67 ± .672
800	16.28 ± 6.28	13.04 ± 3.04	4.42 ± .424
900	14.70 ± 4.70	18.82 ± 8.82	4.70 ± .702

**Table 4:** Relative contribution of environmental variables to the variance of ecological stoichiometry of the *C. fargesii* community.

Order	Variable	Contribution (%)	*F*	*p*
1	Elevation	46.4	8.2	0.002
2	Soil moisture	33.8	8.0	0.002
3	Soil temperature	16.0	4.4	0.006
4	Soil pH	3.9	1.1	0.394

### Relationships between the soil stoichiometry, environmental factors, and plant stoichiometry in the *C. fargesii* population

Soil influences plant growth, productivity, and distribution (Condit et al., 2013) which are relevant to the biogeochemical cycling of nutrients in terrestrial ecosystems (Izquierdo, Houlton & van Huysen, 2013; Tian et al., 2010). Previous studies have demonstrated that soil P was strongly related to leaf N and P (Han et al., 2005; Hedin, 2004). However, we found that soil P showed no relationship with leaf stoichiometry, indicating that soil P did not govern nutrient accumulation in leaves from the study area. Leaf Ca and Mg were found to be negatively correlated with soil K. Previous studies have shown that a high concentration of one cation could cause an imbalance in the concentrations of other cations in the soil (Hailu et al., 2015). Therefore, Ca and Mg absorption by plants was negatively affected by the excess K in the soil (Hailu et al., 2015; Xue et al., 2019). There was a negative relationship between the soil and leaf N, but a previous study showed that soil N was not correlated with leaf N across Chinese grasslands (He et al., 2010). We explored the relative contribution of elevation gradients and soil microenvironment (soil temperature, soil pH, and soil moisture rate) to ecological stoichiometry variation. Our results show that elevation was the most important factor impacting the stoichiometry of *C. fargesii*. However, elevation had the least impact on stoichiometry variation for *Pinus taiwanensis* within our study area, which indicates that this evergreen broad-leaf species is more sensitive than coniferous trees. These results imply that the ecological stoichiometry of *C. fargesii* will alter during global warming.

## Conclusions

Leaf N, K, and Ca levels within *C. fargesii* were significantly related to elevation, while leaf P and Mg showed no relationship with elevation. The leaf N:P indicated that *C. fargesii* was limited by N in this subtropic forest ecosystem in China. Soil P showed no relationship with leaf stoichiometry. The elevation explained nearly half of the variation of ecological stoichiometry in *C. fargesii.* Our results may improve our understanding of the biogeochemical cycle for nutrients in the subtropical forests of China.

##  Supplemental Information

10.7717/peerj.11553/supp-1 Supplemental Information 1 Raw data used in this study. The raw data includes the nutrient element concentration of leaf and soil samples The value of *R*^2^ greater than 0.16 in each plot indicates significant correlation was detected.

10.7717/peerj.11553/supp-2 Supplemental Information 2 Linear regressions between elevation and leaf N, P, K, Ca, and Mg concentrations (A–E) and leaf N:P, N:K, N:Ca, N:Mg, P:K, P:Ca, P:Mg, K:Ca, K:Mg, and Ca:Mg ratios (F–O) The value of *R*^2^ greater than 0.16 in each plot indicates significant correlation was detected.

10.7717/peerj.11553/supp-3 Supplemental Information 3 Linear regressions between elevation and root N, P, K, Ca, and Mg concentrations (A–E) and leaf N:P, N:K, N:Ca, N:Mg, P:K, P:Ca, P:Mg, K:Ca, K:Mg, and Ca:Mg ratios (F–O) The value of *R*^2^ greater than 0.16 in each plot indicates significant correlation was detected.

## Additional Information and Declarations

### Data Availability

The following information was supplied regarding data availability: The raw data are available in the Supplementary File.
