# Supplementary figures and images for: Changes in the stoichiometry of Castanopsis fargesii along an elevation gradient in a Chinese subtropical forest
Source: PeerJ. 2021 Jun 1;9:e11553. doi: 10.7717/peerj.11553 (PMC8176907; doi:10.7717/peerj.11553)

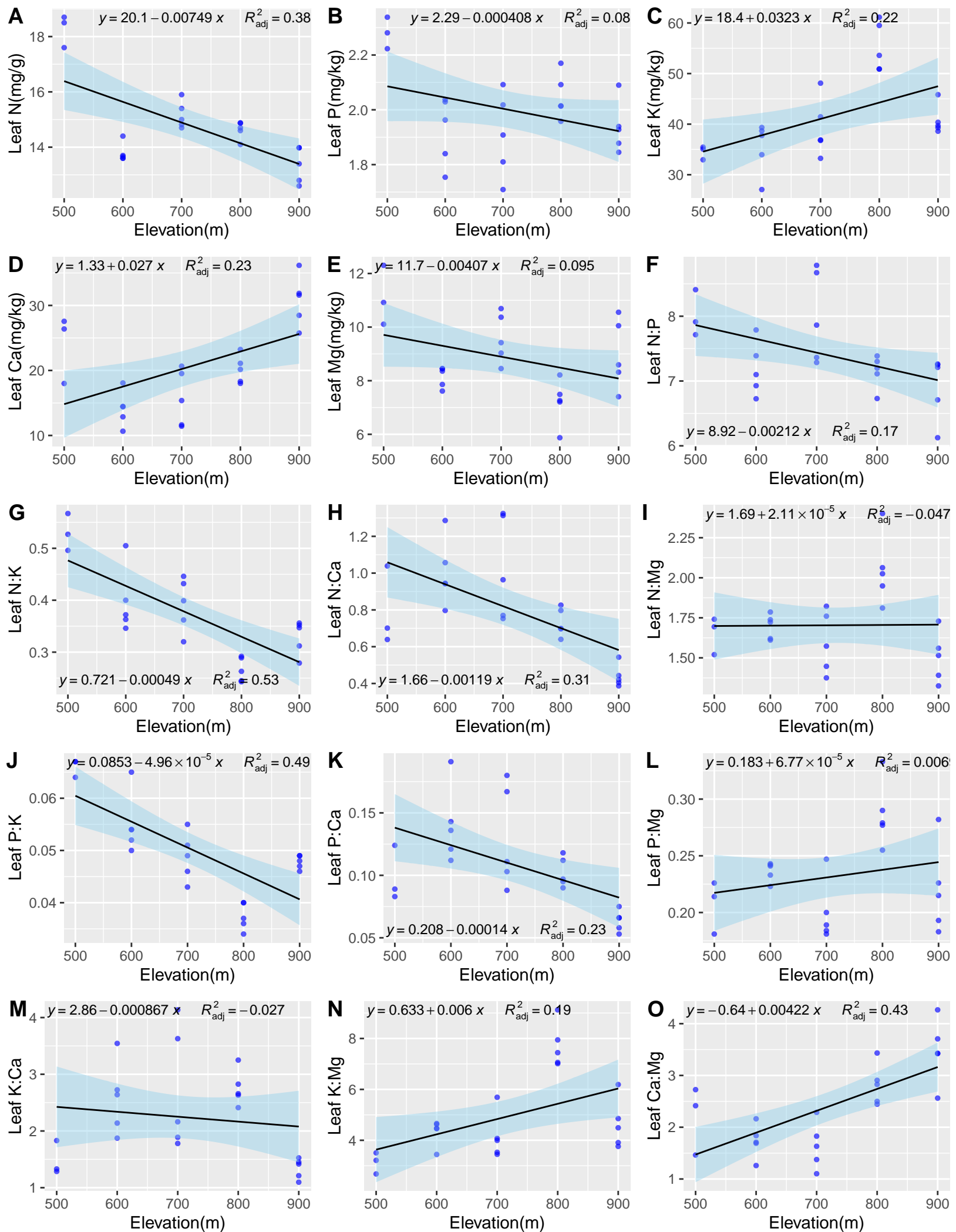

Supplement: Supplemental Information 2 — The value of R2 greater than 0.16 in each plot indicates significant correlation was detected. [file peerj-09-11553-s002.pdf]

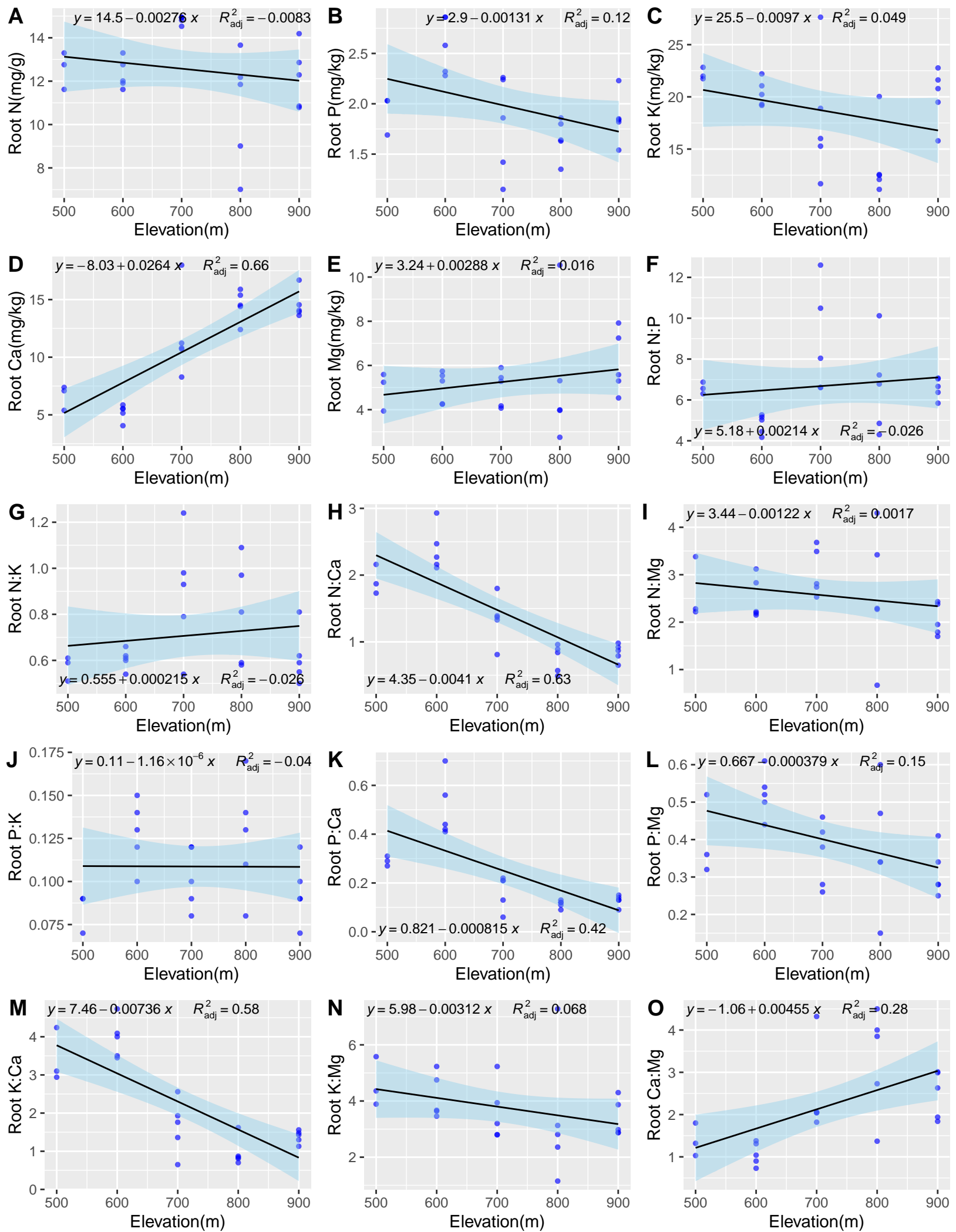

Supplement: Supplemental Information 3 — The value of R2 greater than 0.16 in each plot indicates significant correlation was detected. [file peerj-09-11553-s003.pdf]
